# Supplementary material for: RNF43 inhibits WNT5A-driven signaling and suppresses melanoma invasion and resistance to the targeted therapy
Source: eLife. 2021 Oct 27;10:e65759. doi: 10.7554/eLife.65759 (PMC8550759; doi:10.7554/eLife.65759)
Supplement: Supplementary file 1. [file elife-65759-supp1.pptx]

## Slide 1
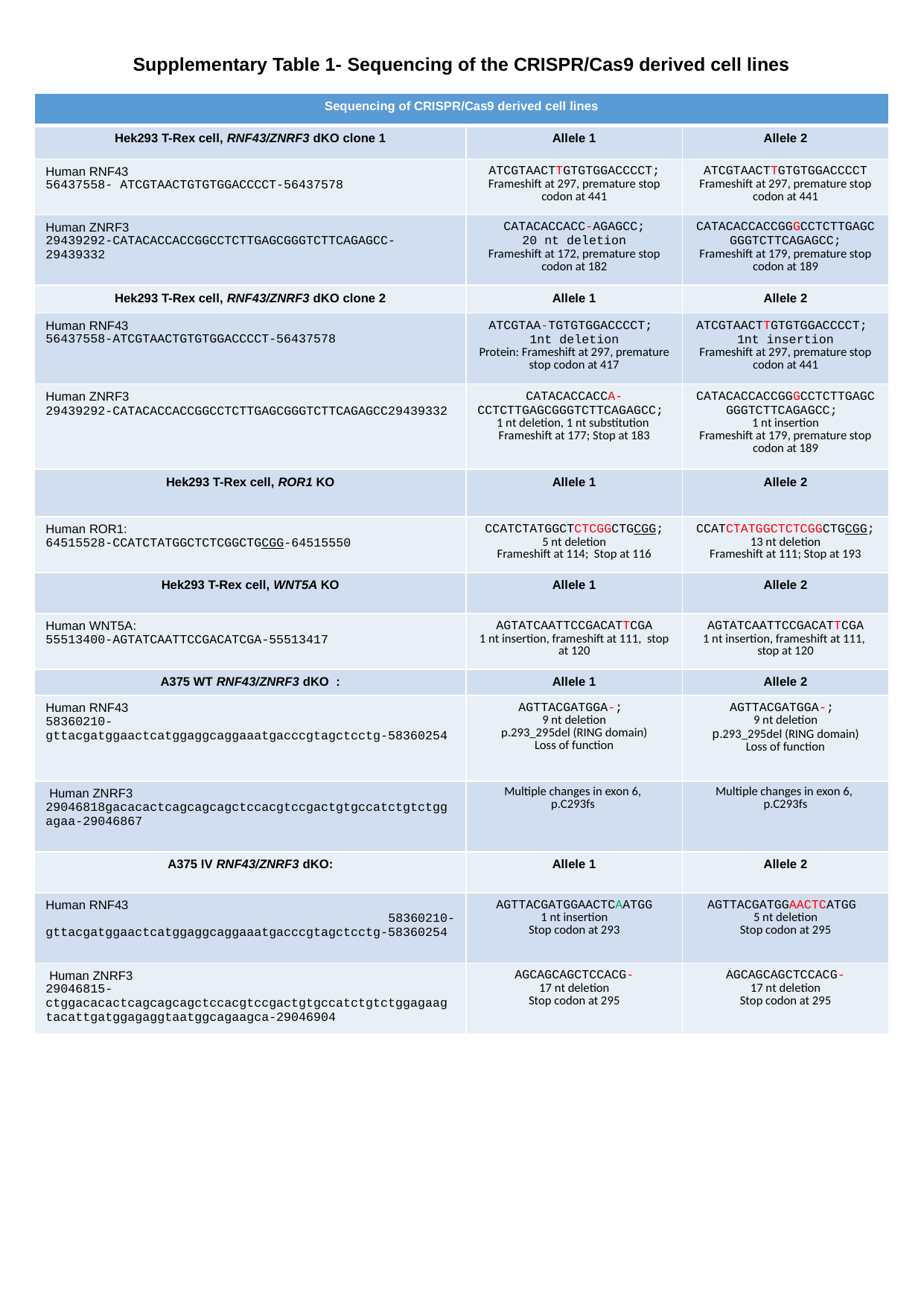

Supplementary Table 1- Sequencing of the CRISPR/Cas9 derived cell lines
| Sequencing of CRISPR/Cas9 derived cell lines | | |
| --- | --- | --- |
| Hek293 T-Rex cell, RNF43/ZNRF3 dKO clone 1 | Allele 1 | Allele 2 |
| Human RNF43 56437558- ATCGTAACTGTGTGGACCCCT-56437578 | ATCGTAACTTGTGTGGACCCCT; Frameshift at 297, premature stop codon at 441 | ATCGTAACTTGTGTGGACCCCT Frameshift at 297, premature stop codon at 441 |
| Human ZNRF3 29439292-CATACACCACCGGCCTCTTGAGCGGGTCTTCAGAGCC-29439332 | CATACACCACC-AGAGCC; 20 nt deletion Frameshift at 172, premature stop codon at 182 | CATACACCACCGGGCCTCTTGAGCGGGTCTTCAGAGCC; Frameshift at 179, premature stop codon at 189 |
| Hek293 T-Rex cell, RNF43/ZNRF3 dKO clone 2 | Allele 1 | Allele 2 |
| Human RNF43 56437558-ATCGTAACTGTGTGGACCCCT-56437578 | ATCGTAA-TGTGTGGACCCCT; 1nt deletion Protein: Frameshift at 297, premature stop codon at 417 | ATCGTAACTTGTGTGGACCCCT; 1nt insertion Frameshift at 297, premature stop codon at 441 |
| Human ZNRF3 29439292-CATACACCACCGGCCTCTTGAGCGGGTCTTCAGAGCC29439332 | CATACACCACCA-CCTCTTGAGCGGGTCTTCAGAGCC; 1 nt deletion, 1 nt substitution Frameshift at 177; Stop at 183 | CATACACCACCGGGCCTCTTGAGCGGGTCTTCAGAGCC; 1 nt insertion Frameshift at 179, premature stop codon at 189 |
| Hek293 T-Rex cell, ROR1 KO | Allele 1 | Allele 2 |
| Human ROR1: 64515528-CCATCTATGGCTCTCGGCTGCGG-64515550 | CCATCTATGGCTCTCGGCTGCGG; 5 nt deletion Frameshift at 114; Stop at 116 | CCATCTATGGCTCTCGGCTGCGG; 13 nt deletion Frameshift at 111; Stop at 193 |
| Hek293 T-Rex cell, WNT5A KO | Allele 1 | Allele 2 |
| Human WNT5A: 55513400-AGTATCAATTCCGACATCGA-55513417 | AGTATCAATTCCGACATTCGA 1 nt insertion, frameshift at 111, stop at 120 | AGTATCAATTCCGACATTCGA 1 nt insertion, frameshift at 111, stop at 120 |
| A375 WT RNF43/ZNRF3 dKO : | Allele 1 | Allele 2 |
| Human RNF43 58360210-gttacgatggaactcatggaggcaggaaatgacccgtagctcctg-58360254 | AGTTACGATGGA-; 9 nt deletion p.293\_295del (RING domain) Loss of function | AGTTACGATGGA-; 9 nt deletion p.293\_295del (RING domain) Loss of function |
| Human ZNRF3 29046818gacacactcagcagcagctccacgtccgactgtgccatctgtctggagaa-29046867 | Multiple changes in exon 6, p.C293fs | Multiple changes in exon 6, p.C293fs |
| A375 IV RNF43/ZNRF3 dKO: | Allele 1 | Allele 2 |
| Human RNF43 58360210-gttacgatggaactcatggaggcaggaaatgacccgtagctcctg-58360254 | AGTTACGATGGAACTCAATGG 1 nt insertion Stop codon at 293 | AGTTACGATGGAACTCATGG 5 nt deletion Stop codon at 295 |
| Human ZNRF3 29046815-ctggacacactcagcagcagctccacgtccgactgtgccatctgtctggagaagtacattgatggagaggtaatggcagaagca-29046904 | AGCAGCAGCTCCACG- 17 nt deletion Stop codon at 295 | AGCAGCAGCTCCACG- 17 nt deletion Stop codon at 295 |
